# Effect of extracellular matrix stiffness on efficacy of Dapagliflozin for diabetic cardiomyopathy

**DOI:** 10.1186/s12933-024-02369-x

**Published:** 2024-07-24

**Authors:** Tong Zhu, Zhaoyang Ye, Jingjing Song, Junjie Zhang, Yuxiang Zhao, Feng Xu, Jun Wang, Xin Huang, Bin Gao, Fei Li

**Affiliations:** 1 The Key Laboratory of Biomedical Information Engineering of Ministry of Education, School of Life Science and Technology, Xi’an Jiaotong University, Xi’an, 710049 P.R. China; 2 Bioinspired Engineering and Biomechanics Center (BEBC), Xi’an Jiaotong University, Xi’an, 710049 P.R. China; 3 Department of Cardiovasology, Xidian Group Hospital, Xi’an, 710077 P.R. China; 4 Department of Health Evaluation and Promotion, The First Affiliated Hospital of Xi’an Jiaotong University, Xi’an, 710061 P.R. China; 5 Department of Cardiology, The First Affiliated Hospital of Xi’an Jiaotong University, Xi’an, 710061 P.R. China; 6 Department of Endocrinology, Tangdu Hospital, Air Force Military Medical University, Xi’an, 710032 P.R. China

**Keywords:** Diabetic cardiomyopathy, Mechanical microenvironment, Mechanomedicine, AT_1_R, FAK, NOX2

## Abstract

**Background:**

Extracellular matrix (ECM) stiffness is closely related to the progress of diabetic cardiomyopathy (DCM) and the response of treatment of DCM to anti-diabetic drugs. Dapagliflozin (Dapa) has been proven to have cardio-protective efficacy for diabetes and listed as the first-line drug to treat heart failure. But the regulatory relationship between ECM stiffness and treatment efficacy of Dapa remains elusive.

**Materials and methods:**

This work investigated the effect of ECM stiffness on DCM progression and Dapa efficacy using both in vivo DCM rat model and in vitro myocardial cell model with high glucose injury. First, through DCM rat models with various levels of myocardial injury and administration with Dapa treatment for four weeks, the levels of myocardial injury, myocardial oxidative stress, expressions of AT_1_R (a mechanical signal protein) and the stiffness of myocardial tissues were obtained. Then for mimicking the stiffness of myocardial tissues at early and late stages of DCM, we constructed cell models through culturing H9c2 myocardial cells on the polyacrylamide gels with two stiffness and exposed to a high glucose level and without/with Dapa intervention. The cell viability, reactive oxygen species (ROS) levels and expressions of mechanical signal sensitive proteins were obtained.

**Results:**

The DCM progression is accompanied by the increased myocardial tissue stiffness, which can synergistically exacerbate myocardial cell injury with high glucose. Dapa can improve the ECM stiffness-induced DCM progression and its efficacy on DCM is more pronounced on the soft ECM, which is related to the regulation pathway of AT_1_R-FAK-NOX2. Besides, Dapa can inhibit the expression of the ECM-induced integrin β1, but without significant impact on piezo 1.

**Conclusions:**

Our study found the regulation and effect of biomechanics in the DCM progression and on the Dapa efficacy on DCM, providing the new insights for the DCM treatment. Additionally, our work showed the better clinical prognosis of DCM under early Dapa intervention.

**Supplementary Information:**

The online version contains supplementary material available at 10.1186/s12933-024-02369-x.

## Introduction

Diabetic cardiomyopathy (DCM), which is dysregulated glucose and lipid metabolism normally caused by insulin resistance, is a major cause of morbidity and mortality in the world, and shows a continuous increasing trend with the rises in obesity and type 2 diabetes (T2D) [1, 2]. The hallmark of DCM is the left ventricular hypertrophy and dysfunction, progressing to overt heart failure accompanied by reduced systolic function in later phases [3]. The molecular mechanisms about the pathological changes in diabetic hearts includes many factors, including oxidative stress, enhanced advanced glycation end products (AGEs), mitochondrial dysfunction, inflammation, and cell death [4]. Epidemiological statistics shows that approximately one-quarter of diabetic patients worldwide have myocardial lesions and the cardiovascular disease accounts for 50% of deaths, imposing a tremendous burden on society and family economics [5]. Therefore, it is urgently needed to develop effective therapeutic methods for DCM.

Dapagliflozin (Dapa), which is a selective sodium-glucose co-transporter-2 inhibitor, has been widely used for T2D treatment [6, 7]. Recently, in the 2021 European Society of Cardiology guidelines for diagnosis and treatment of acute and chronic heart failure, the use of SGLT2 has been listed as a standard treatment strategy for heart failure [8]. The large-scaled randomized trials of SGLT2 inhibitors have been reported to decrease the cardiovascular complications and events in T2D patients with heart failure [9]. Moreover, in the Dapa-heart failure trials in the patients with heart failure with decreased ejection fraction (HFrEF), the SGLT2 inhibitor (e.g., Dapa) presents an obvious cardiovascular benefit, regardless of patients with/without T2D [10]. Despite the profound interests in the Dapa treatment for DCM, the efficacy of Dapa in ameliorating DCM and its pathophysiological mechanisms remain elusive.

Extracellular matrix (ECM) in cell microenvironment provides both structural supports for cells and biochemical and biophysical cues to regulate cell behaviors. In T2D, enhanced expressions of ECM proteins (e.g., elastin, collagen, laminin and fibronectin) induced by chronic hyperglycemia result in the increased ECM stiffness, which could affect the cardiac pumping function [11] and structure [12]. Recent studies showed that stiffened ECM can accelerate the DCM progress [13], inducing myocardial cell hypertrophy and myofibroblast activation, which are relevant to fibrosis and inflammatory reaction [14, 15]. Besides, changes in tissue mechanics can also lead to differences in responsiveness of cardiovascular drugs by regulating the biological behavior of cells [16, 17]. It can thus be inferred that biochemical effects of Dapa and biomechanics of ECM are highly relevant with the context of DCM responsiveness to the efficacy of anti-diabetic medicine. But the influence of ECM stiffness on DCM progression and efficacy of anti-diabetic drugs remains elusive. The study of the relationship between ECM stiffness and treatment efficacy of anti-diabetic drugs can attribute to a better understanding of DCM pathogenesis and identification of improved therapeutic strategies.

**Scheme 1:**
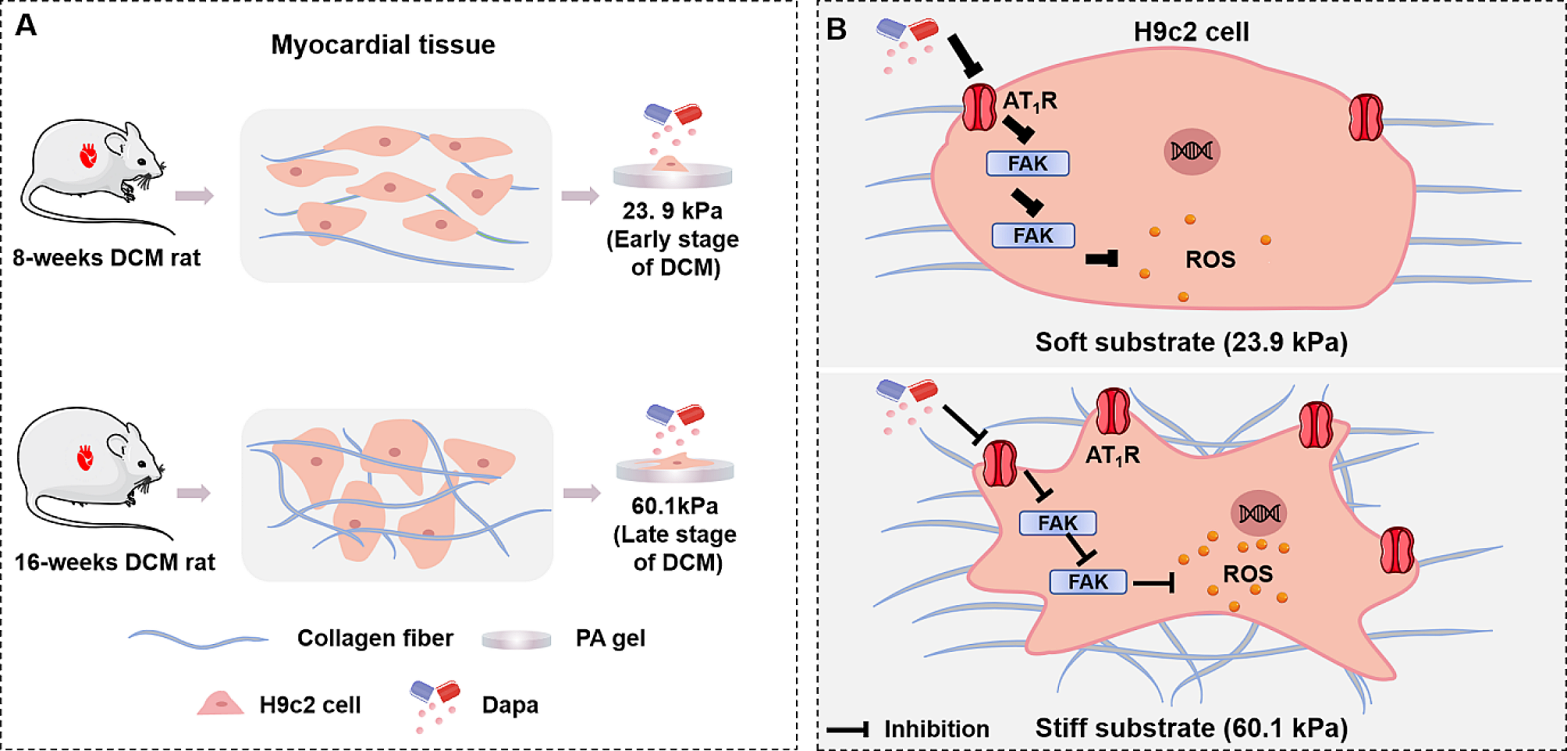
Schematic diagram of Dapa efficacy on DCM under different myocardial tissue stiffness. **A** Construction of culture substrate for H9c2 myocardial cells in vitro by simulating the stiffness of myocardial tissues in rats with different degrees of DCM. **B** Dapa inhibits the progression of DCM through the regulatory pathway of AT_1_R-FAK-NOX2 on ECM with different stiffness

In this work, we investigated the therapeutic influence of ECM stiffness on efficacy of Dapa for DCM treatment. As shown in Scheme 1A, firstly, we constructed the rat models at the early and late stages of DCM and treated them with Dapa, from which we obtained the stiffness of cardiac tissues of the rat models. Then, we constructed cell models via culturing myocardial cells on the polyacrylamide (PA) gels with stiffness mimicking the stiffness of myocardial tissues at early and late stages of DCM with/without Dapa intervention. The therapeutic mechanism of Dapa on DCM rats and the therapeutic efficacy of Dapa were studied by comparing the cardiac function, myocardial cell viability and oxidative stress before and after Dapa intervention through in vivo and in vitro models. According to the characterization results of the mechanical signal proteins, the underlying mechanism of Dapa efficacy on DCM under different ECM stiffness were obtained. Our study proved the positive effect of Dapa on DCM treatment by inhibiting the expressions of mechanical signaling proteins (i.e., angiotensin II type 1 receptor (AT_1_R)), which reduces the downstream expressions of focal adhesion kinase (FAK) and NADPH oxidase 2 (NOX2) and leads to the decreased production of reactive oxygen species (ROS) (Scheme 1B), and Dapa had a more significant therapeutic effect on DCM than those under stiffer ECM conditions. This findings could propose a novel therapeutic target for DCM treatment and contribute to the understanding of Dapa efficacy to DCM from a biopharmacological perspective.

## Methods

### Animals

Non-insulin-dependent spontaneous type 2 diabetes rats (Goto-Kakizaki Rats, GK rats), which were developed into diabetes cardiomyopathy [18, 19], were used as the animal models in this work. The male GK rats (14 weeks old and weighing of 200–250 g) with average blood glucose levels above 11.1 mmol/L were provided by Carvens Laboratory Animal Co., Ltd. (Changzhou, China). All the rats were fed with high-fat diet and performed with echocardiographic cardiac function measurements every two weeks. Dapagliflozin (0.1 mg/kg/day, MedChemExpress, New Jersey, USA) was administered to the rats for a duration of four weeks prior to euthanasia. The GK rats were randomly divided in five groups: control group (no T2D), 8-weeks group (primary diabetes cardiomyopathy rats), 16-weeks group (late diabetes cardiomyopathy rats), 8-weeks received Dapa group, and 16-weeks received Dapa group. All the rats were anesthetized by injection with 2% pentobarbital sodium. The in vivo experiments and animal administration steps were carried out following the NIH Guideline for Care and Application of Laboratory Animals.

### Echocardiographic evaluation of rats

An AM-mode echocardiography configured with a 17.5 MHz linear array transducer system (iE33, Philips, Massachusetts, USA) was applied to measure the cardiac structure and function, including the left ventricular internal diameter at end-diastole (LVIDd, mm), left ventricular posterior wall thickness at end-diastole (LVPWd, mm), interventricular septum thickness at end-diastole (IVSTd, mm), ejection fraction (EF, %), and fractional shortening (FS, %). The rats were euthanized and their blood samples and heart tissues were then collected.

### Characterization of Young’s modulus of myocardial tissues

The fresh myocardial tissues of rats were firstly cut into slices with a thickness of 500 μm, and fixed on a glass slide with immersing them into phosphate buffered saline (PBS) for less than 10 min. Then the stiffness of the sliced myocardial tissues (Young’s modulus, *E*) were measured immediately by a Nanoindenter instrument (Piuma, Optics11, Amsterdam, Netherlands) with a probe with radius of 47 μm and cantilever stiffness of 0.5 N/m. The Young’s modulus (*E*) of myocardial tissues were calculated using the Hertzian model and obtained through fitting the recorded loading curves to the following equation (Eq. (1)).

1 $$\:E=\frac{P*3/4}{\sqrt{R}\cdot\:{{h}_{t}}^{3/2}}$$

where *P* is the load in the peak of fit, *R* is the tip radius, and *h*_*t*_ represents the indentation depth [20, 21].

### Measurements of brain natriuretic peptide (BNP) and cardiac troponin T (c-TnT)

The enzyme-linked immunosorbent assay (ELISA) kits (Solarbio, Beijing, China) were used to measure the brain natriuretic peptide (BNP) and cardiac troponin T (c-TnT) in rat serums following the producer’s instructions. The absorbance of plasma samples at 450 nm was recorded by a Spark 10 M Multimode Microplate Reader (TECAN, Switzerland).

### Masson’s trichrome and immunohistochemistry staining

To observe the extracellular collagen deposition of myocardial tissues, the myocardial tissues fixed in 4% paraformaldehyde (Beyotime, Shanghai, China) were embedded in paraffin, followed by sectioning at 5 µm and deparaffinized and staining with a Masson’s trichrome staining kit (Servicebio, Wuhan, China). Then, immunohistochemistry staining was utilized to characterize the expressions of AT_1_R, p-FAK, FAK and NOX2 of myocardial tissue slices, respectively. The deparaffinized slices were kept in a solution containing 10 mM sodium citrate (pH 6.0) at 100 ℃ for 10 min. After blocking the nonspecific binding sites of myocardial tissue slices by 8% goat serum, the slices of myocardial tissues were incubated with the following primary antibodies at 4℃ overnight: anti-focal adhesion kinase (phospho Y397) antibody (FAK, ab81298, Abcam, Cambridge, MA, USA), anti-focal adhesion kinase antibody (FAK, ab40794, Abcam, Cambridge, MA, USA), anti-angiotensin II type-1 receptor antibody (AT_1_R, 25343-1-AP, Proteintech, Wuhan, China) and NOX2 rabbit polyclonal antibody (Servicebio, Wuhan, China). Then, the slices were incubated with the corresponding secondary antibody under room temperature for 2.5 h, and after washing the slices with PBS for three times, 3,3’-diaminobenzidine tertrahydrochloride (DAB) horseradish peroxidase color development kit (Beyotime, Shanghai, China) was employed for chromogenic reaction. Microphotographs of myocardial tissue slices were recorded and analyzed with an inverted fluorescence microscope (Olympus IX81, Tokyo, Japan).

### Preparation and characterization of polyacrylamide (PA) gels

A procedure described in the prior literatures was used to prepare the PA gels for culturing H9c2 cells [22, 23]. Briefly, we firstly prepared 40%(w/v) acrylamide monomers (Macklin, Shanghai, China), 2%(w/v) N, N-methylene-bis-acrylamide (MBA) (Macklin), 10%(w/v) ammonium persulfate (APS, Sigma-Aldrich) and tetramethylethylenediamine (TEMED, Sigma-Aldrich). For the preparation of the PA gels with stiffness around 24.0 and 60.0 kPa, the mass/volume concentrations of APS and TEMED were 1% and 0.1% and the ratios of acrylamide (%)/MBA (%) were 10/0.29 and 12/0.25, respectively. Then, 50 µL of the prepared polymer solution was dropped onto the hydrophilic-treated glass bottom of a petri dish with covering with a dichlorodimethylsilane treated 18 mm-in-diameter glass coverslip. Followed by polymerization of the PA gels, the top coverslips were peeled off with removing the remaining monomers and cross-linkers though PBS washing. Finally, 1 mg mL^−1^ of the cross-linker N-sulfosuccinimidyl 6-(4’-azido-2’-nitrophenylamino) hexanoate (Sulfo SANPAH, Thermo Scientific, Waltham, MA, USA) was added into the PA gels and photoactivated by ultraviolet light of 365 nm exposure for 10 min. Then the PA gels were washed with 50 mM HEPES (pH 8.5) and kept in a solution containing 50 µg mL^−1^ rat tail tendon collagen type I (Corning, Manassas, VA, USA) overnight. The same nanoindenter instrument (Piuma, Optics11, Amsterdam, Netherlands) was used to measure the Young’s modulus of the PA gels. The Young’s modulus (*E*) of the PA gels were obtained through fitting the recorded loading curves to Eq. (1). Before seeding cells, the as-prepared PA gels were sterilized by ultraviolet light at 245 nm in an ultraclean cabinet for 2 h.

### Cell culture

H9c2 cells (iCell-r012, Shanghai, China) were cultured in DMEM media (Corning, Manassas, VA, USA) containing 10% (v/v) fetal bovine serum (FBS, Gibco, Grand Island, NY, USA) and 1% (v/v) penicillin/streptomycin (Sigma, St. Louis, MO, USA) in a 5% CO_2_ thermostatic incubator at 37℃ for five days. The culture conditions with glucose concentrations of 5.5 mmol/L and 33 mmol/L were prepared and used to mimic the in vivo low glucose (LG) and high glucose (HG) DCM conditions. For the Dapa-treated group, the H9c2 cells were cultured with addition of 0.5 µM Dapa for 24 h.

### Cell viability

The H9c2 cells were seeded on the PA gels with different stiffness at a density of 1 × 10^5^ cells and then cultured with LG, HG conditions or Dapa treatment. After removing the original medium, a fresh medium containing CCK8 solution was added into the petri dish and then incubated in dark at 37℃ for 2 h according to the manufacturer’s instruction of Cell Count Kit-8 (Beyotime, Shanghai, China). A Spark 10 M Multimode Microplate Reader (TECAN, Switzerland) was used to record the absorbance at 450 nm.

### Immunofluorescence staining

The H9c2 cells on the PA gels were first fixed with 4% paraformaldehyde for 10 min and followed by permeabilization with 0.5% Triton X-100 (Sigma-Aldrich, St. Louis, MO, USA) for 15 min. The cells were blocked with 10% goat serum (Gibco, Grand Island, NY, USA) at room temperature for 2 h. Then, the H9c2 cells were incubated with the following primary antibodies at 4℃ overnight: anti-focal adhesion kinase (phospho Y397) antibody (FAK, ab81298, Abcam, Cambridge, MA, USA), anti-focal adhesion kinase antibody (FAK, ab40794, Abcam, Cambridge, MA, USA), anti-angiotensin II type-1 receptor antibody (AT_1_R, 25343-1-AP, Proteintech, Wuhan, China), NOX2 rabbit polyclonal antibody (Servicebio, Wuhan, China), Piezo1 antibody (DF12083, Affinity, Jiangsu, China) and CD29 (Integrin β1) monoclonal antibody (13-0291-80, Thermo Fisher Scientific, Waltham, MA, USA). Then, the secondary antibodies (Alexa Fluor-488 goat anti-mouse antibody (Ab150077, Abcam) or Alexa Fluor-594 goat anti-rabbit antibody (Ab150116, Abcam) were incubated with H9c2 cell samples at room temperature for 2 h. For staining cell nuclei, DAPI (D9542, Sigma-Aldrich, St. Louis, MO, USA) was incubated with H9c2 cells for 10 min before fluorescence photography. A laser scanning confocal microscope (FV3000 Olympus, Tokyo, Japan) was employed to record the fluorescence images of H9c2 cells.

### Measurement of intracellular ROS levels

The measurement of intracellular ROS levels was performed with a ROS detection assay kit (ab139476, Abcam, Cambridge, MA, USA). The H9c2 cells were incubated with the fluorescent dye at 37 °C for 30 min following the manufacture’s instruction. The fluorescence images of cells were recorded by a laser scanning confocal microscope (FV3000 Olympus, Tokyo, Japan).

### Western blotting

All the cell samples were lysed with a RIPA lysis buffer (Solarbio, Beijing, China). The total protein concentrations of H9c2 myocardial cells were measured by a bicinchoninic acid (BCA) protein assay kit (Beyotime, Shanghai, China). The protein samples were separated by sodium dodecyl sulfate-polyacrylamide gel (SDS-PAGE) and transferred to polyvinylidene difluoride (PVDF) membranes (Millipore Bedford, MA, USA). The primary antibodies (AT_1_R, t-FAK, p-FAK, NOX2) were added on the PVDF membranes with specific targeted protein molecular weight for incubation overnight. After TBST rinsing, the horseradish peroxidase-conjugated secondary antibodies were added on the PVDF membranes for 1 h incubation. The proteins blots were characterized with a chemiluminescence imaging system (ChemiScope 3300 mini, Shanghai, China) and analyzed with Image J software.

### Statistical analysis

GraphPad Prism 9 (GraphPad Software, La Jolla, CA, USA) was used to statistically analyze the experimental data. Statistics are shown as the mean ± standard error of the mean for all quantitative data, with *n* = 4 for the animal tests and *n* ≥ 3 for the cell tests. Two-way ANOVA was utilized to evaluate statistical significance, followed by post-hoc Tukey test and paired *t* test, respectively (ns, no significant difference, *p < 0.05, **p < 0.01, ***p < 0.001, and ****p < 0.0001).

## Results

### Dapa reduced myocardial tissue stiffness and collagen protein expressions in DCM rats

**Fig. 1:**
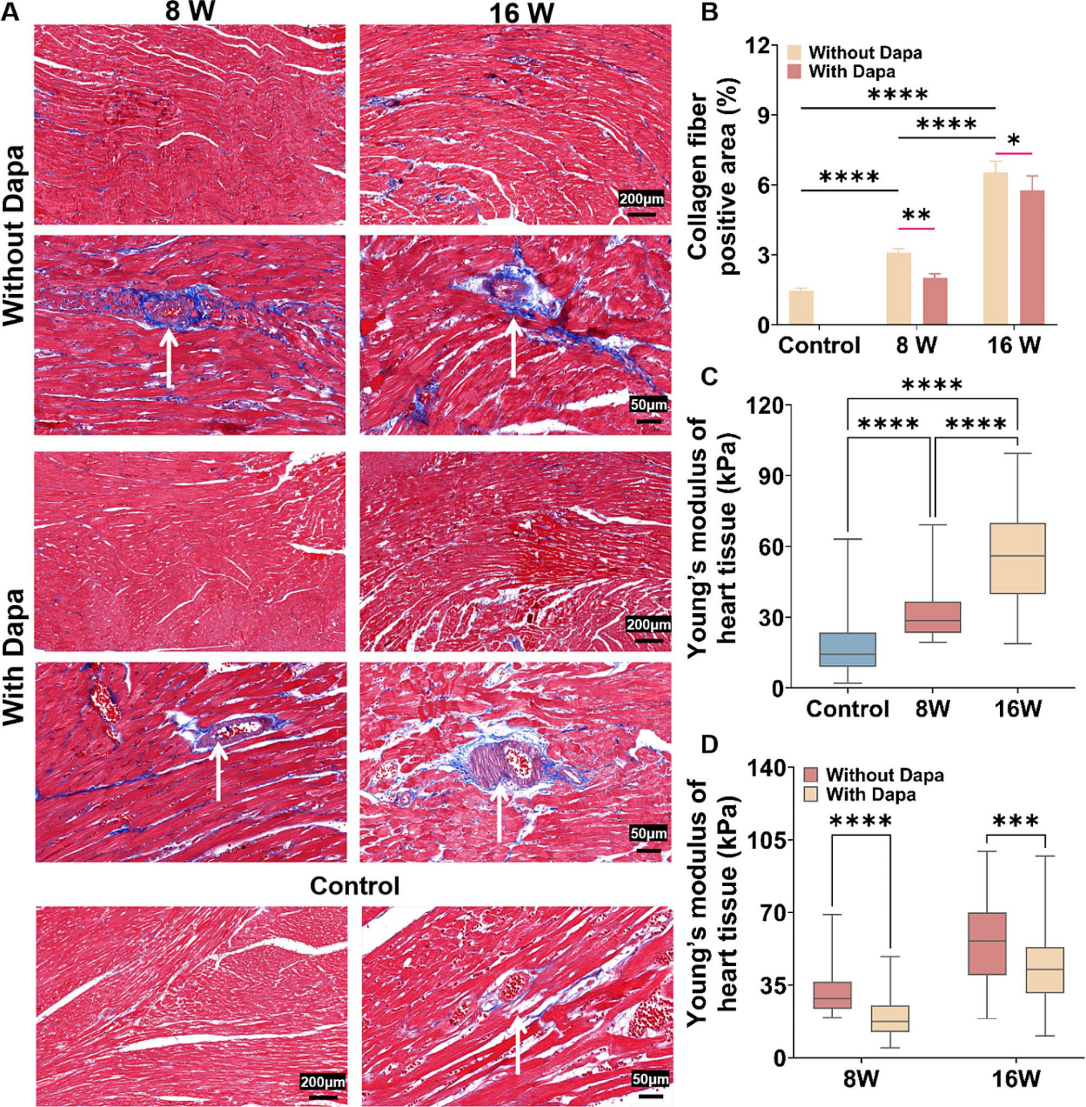
Characterization results of the collagen protein expressions and myocardial tissue stiffness of the DCM rats. **A** Representative Masson’s trichrome staining images of myocardium without and with Dapa treatment. **B** Statistical histograms of collagen fiber-positive area (%) (*n* = 4). **C** Statistics of the myocardial tissue stiffness of DCM rats in the control group, 8-weeks and 16-weeks groups without Dapa treatment (*n* = 4). **D** Statistics of the myocardial tissue stiffness of DCM rats in the 8-weeks and 16-weeks groups without and with Dapa treatment (*n* = 4). *p < 0.05, **p < 0.01, and ****p < 0.0001 determined by two-way ANOVA

To explore the mechanical characteristics of heart tissue before and after Dapa treatment at different degrees of DCM, we firstly constructed in vivo DCM rat models and characterized the myocardial tissue stiffness and collagen contents of rats. From Fig. **S1**, we observed no obvious difference in the heart weights among the 8-weeks, 16-weeks and Dapa groups, indicating that the animal experimental conditions were consistent and these groups can thus be used for the subsequent experiments. We observed that compared to the control group without diabetes, the collagen contents in the myocardial tissues increased with the progression of diabetes (Fig. 1A), indicating the successful construction of the rats models at different stages of DCM and the Dapa administration could notably attenuate the collagen contents in myocardial tissues. In addition, the Dapa treatment resulted in greater reduction of the collagen contents in the 8-weeks group than the 16-weeks group (Fig. 1B), indicating that Dapa had a different inhibitory effect on the fibrosis progression in rats.

We then measured the myocardial tissue stiffness of DCM rats and defined the myocardial tissue stiffness of DCM rats in the 8-weeks group as soft tissue stiffness, and the myocardial tissue stiffness of DCM rats in the 16 weeks group as stiff tissue stiffness. For the myocardial tissues of the DCM rats, their stiffness increased with the progression of diabetes compared with the control group without diabetes (Fig. 1C). Compared to those groups without Dapa treatment, the tissue stiffness of the Dapa-treated rats decreased (Fig. 1D), indicating that the Dapa treatment can reduce the stiffness of myocardial tissues in the DCM rats, and there are significant differences in the decrease degrees of the stiffness of myocardial tissues between the 8-weeks and 16-weeks groups after the Dapa treatment.

### Dapa delayed T2D progression and ameliorated heart damage in DCM rats

**Fig. 2:**
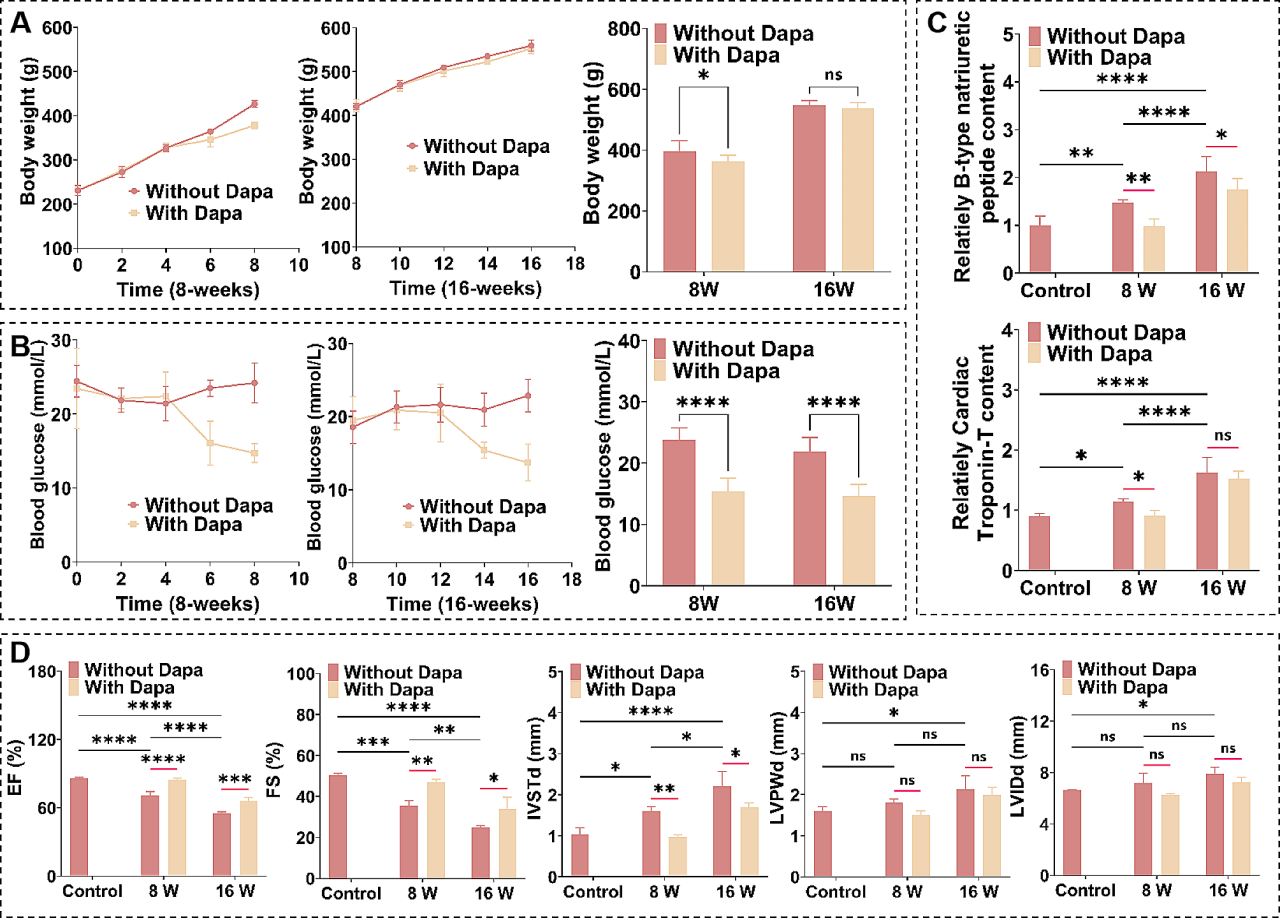
Characterization results of the body weights, blood glucose levels, cardiac function and myocardial injury markers of the DCM rats. **A** Body weights and **B** blood glucose levels of the DCM rats without and with Dapa treatment (*n* = 4). Statistical histogram of **C** the relative levels of BNP and c-TnT in the plasma and **D** echocardiographic statistics of the DCM rats without and with Dapa treatment (*n* = 4). ns, no significant difference, *p < 0.05, **p < 0.01, ***p < 0.001, and ****p < 0.0001 determined by two-way ANOVA

To study of the efficacy of Dapa in the DCM rats with myocardial tissues with different mechanical properties, we characterized the body weight, blood glucose, cardiac function and myocardial injury markers of the DCM rats. The body weights of the rats showed a continuous increase with the diabetes progression, while the increasing trend attenuated after Dapa administration (Fig. 2A). The 8-weeks group presented a greater degree of body weight loss than the 16-weeks group. The blood glucose levels of the DCM rats were obviously higher than normal blood glucose levels, but did not show an increasing trend with the progression of DCM, and the Dapa treatment decreased the blood glucose levels. Although the decreasing trend of blood glucose levels was more significant in the 8-weeks group than the 16-weeks group, no obvious difference in the decline degrees between these two groups (*p* < 0.0001) was observed (Fig. 2B).

We further characterized the markers of myocardial injury. From the results of brain natriuretic peptide (BNP) and cardiac troponin T (c-TnT) in the serum of rats (Fig. 2C), we can see that compared with the control group, the BNP and c-TnT levels in the serum of the DCM rats were substantially elevated with the development of DCM. Compared to the groups without Dapa treatment, the Dapa groups presented remarkable decreases in the BNP and c-TnT levels, and the 8-weeks group had higher decrease values compared to those of the 16-weeks groups. Moreover, from the characterization results of the indicators of ejection fraction (EF), fractional shortening (FS), interventricular septum thickness at end-diastole (IVSTd), left ventricular posterior wall thickness at end-diastole (LVPWd) and left ventricular internal diameter at end-diastole (LVIDd) of DCM rats (Fig. 2D), the DCM rats exhibited the decreased EF and FS and the increased IVSTd than those of the control group without T2D. Despite no statistical diversity in the LVPWd and LVIDd values between the 8-weeks group and the control group, the 16-weeks group had significant increased LVPWd and LVIDd values than those of the control group. The results of the cardiac function indicators show that with the progression of DCM, the cardiac pumping function decreased, while the cardiac wall thickness increased accompanied with the enlargement of the cardiac cavity. Compared with the groups without Dapa treatment, the Dapa-treated groups had the increased EF and FS values and the decreased IVSTd values. And the improvement degrees of the cardiac function and IVSTd in the DCM rats with stiffer myocardial tissues were lower than those of the DCM rats with softer myocardial tissues. But there was no significant statistical variance in the LVPWd and LVIDd values between the 8-weeks and 16-weeks groups with or without Dapa treatment. All these indicate that Dapa can ameliorate cardiac function and mitigate myocardial injury in the DCM rats, but the amelioration degree of the cardiac function and heart injury in the DCM rats with stiffer myocardial tissues were lower than those of the DCM rats with softer myocardial tissues.

### Dapa inhibited the expression of AT_1_R-FAK-NOX2 pathway in the myocardial tissues of DCM rats

**Fig. 3:**
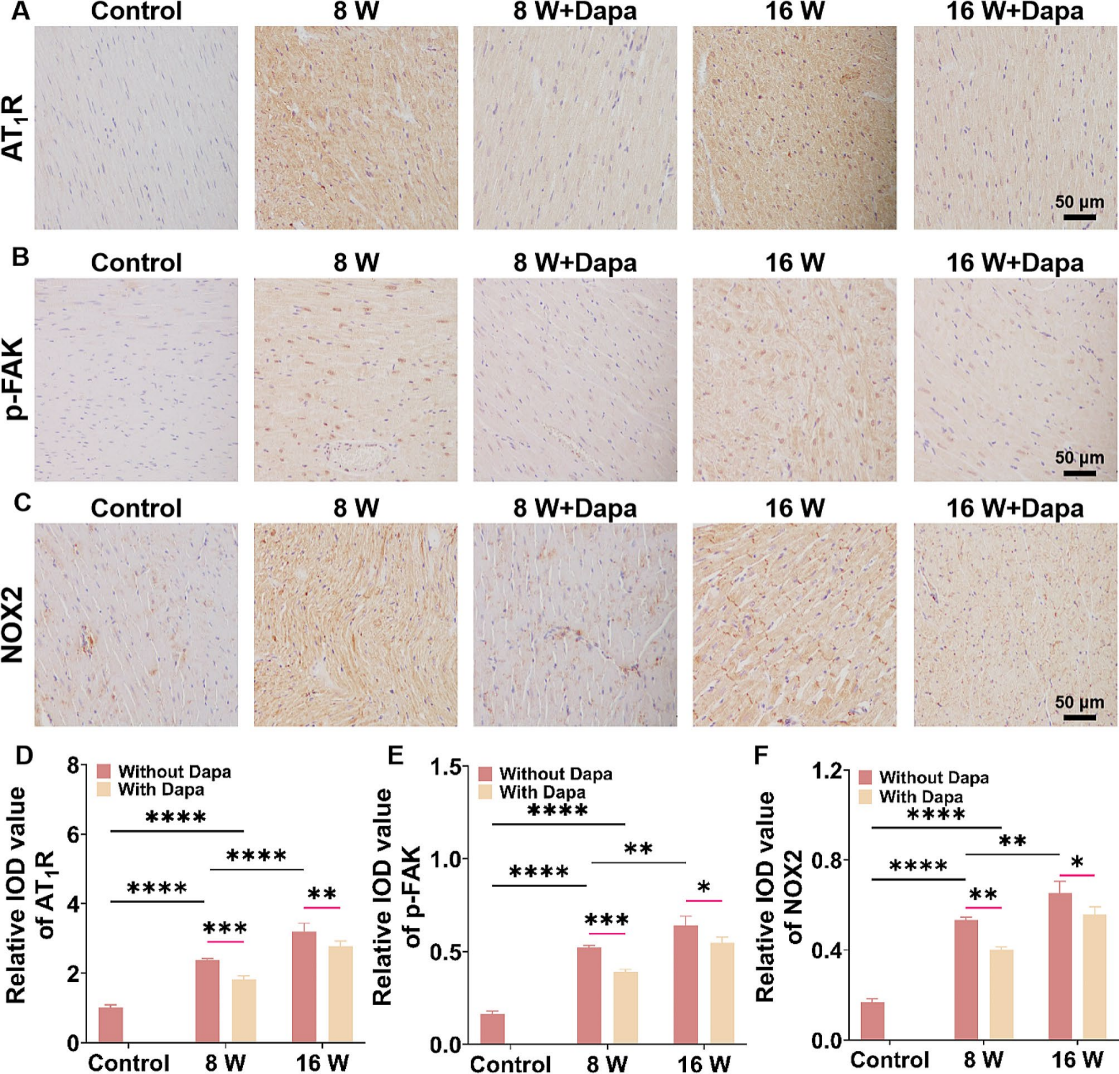
Characterization results of the AT_1_R-FAK-NOX2 expression of the myocardial tissues of DCM rats. **A** AT_1_R, **B** p-FAK and **C** NOX2 immunohistochemical images of the myocardium of DCM rats without and with Dapa treatment. Statistical histograms of the relative **D** AT_1_R, **E** p-FAK and **F** NOX2 contents of the myocardium of DCM rats (*n* = 4). *p < 0.05, **p < 0.01, ***p < 0.001, and ****p < 0.0001 determined by two-way ANOVA.

Since hyperglycemia can result in the elevated oxidative stress level and ECM stiffness in myocardial tissues, the changes in the oxidative stress levels and the mechanical signal pathway in the myocardial tissues of the DCM rats before and after Dapa treatment were characterized. First, we characterized the expressions of NOX2 (the major source of ROS), AT_1_R and p-FAK (two key mechanotransduction proteins) of the myocardial tissues of the diabetes cardiomyopathy rats without and with Dapa intervention. Figure 3A–C show that the expressions of AT_1_R, p-FAK and NOX2 of the rat myocardial tissues increased with the progression of DCM. After treating the DCM rats with Dapa for four weeks, the obvious changes in the expressions of AT_1_R, p-FAK and NOX2 of the myocardial tissues after Dapa intervention were obtained. The Dapa-treated DCM rats had substantial decreases in the expressions of AT_1_R, p-FAK and NOX2 compared to the DCM rats without Dapa treatment. According to the statistical analysis of the expressions of AT_1_R, p-FAK and NOX2 (Fig. 3D–F), a greater decrease degree of the protein expressions in the 8-weeks group than those in the 16-weeks group after Dapa treatment was obtained. These results show that the Dapa intervention can inhibit the expression of the AT_1_R-FAK-NOX2 pathway, and the inhibition degree of the AT_1_R-FAK-NOX2 pathway in the DCM rats with softer myocardial tissues was higher than those of the DCM rats with stiffer myocardial tissues. These animal results indicate that the DCM rats with softer myocardial tissue stiffness had higher improvements in the body weight, blood sugar level, cardiac function and oxidative stress level than those of the DCM rats with stiffer myocardial tissue stiffness, which infers that the Dapa efficacy to DCM varies significantly under different myocardial tissue stiffness conditions. Therefore, we consider that the choice of Dapa intervention time has a significant impact on the clinical prognosis of DCM.

### Dapa ameliorated the viability of H9c2 cells under different mechanical microenvironment

To further investigate the Dapa efficacy on the DCM rats with different myocardial tissue stiffness, an in vitro DCM model was constructed through culturing H9c2 myocardial cells on the PA gels with Young’s modulus of 23.9 kPa and 60.1 kPa (Fig. S2), which were obtained from the myocardial tissues of the early and late stages of DCM rats under a high glucose environment. We also measured the stiffness of the PA gels stiffness after seeding myocardial cells, which shows no significant difference in the PA gels stiffness after cell seeding (Fig. S3). And considering that our in vivo experiments have shown no statistically significant difference in the blood glucose levels between the 8-weeks and 16-weeks DCM rat groups before Dapa treatment, we used a standard high-glucose condition of 33 mmol/L to induce the myocardial injury to establish the in vitro cell model of DCM. From Fig. S4, the cell viability of the H9c2 cells on the PA gels without Dapa treatment decreased with the increased stiffness from 23.9 to 60.1 kPa. And the cell viability attenuation caused by high glucose was significantly improved after Dapa treatment. More importantly, we compared the cell viabilities on the PA gels with various stiffness after Dapa intervention, and found that the improvement degrees of the cell viability were more obvious on the soft ECM compared to those of on the stiff ECM.

### Dapa reduced the expressions of mechanical signal proteins and oxidative stress levels in H9c2 cells under different mechanical microenvironment

From the above immunohistochemical results of myocardial tissues of DCM rats, we can obtain that the expressions of the mechanical signal proteins (e.g., AT_1_R and FAK) and NOX2 increased with the progress of DCM and decreased after Dapa treatment. To further explore the mechanism of mechanical regulation on Dapa efficacy and clarify the Dapa efficacy on the expression of mechanical signal protein and oxidative stress level of H9c2 cells under different mechanical microenvironment, the expressions of AT_1_R, FAK, NOX2 and ROS of the H9c2 cells on the PA gels with various stiffness were measured.

**Fig. 4:**
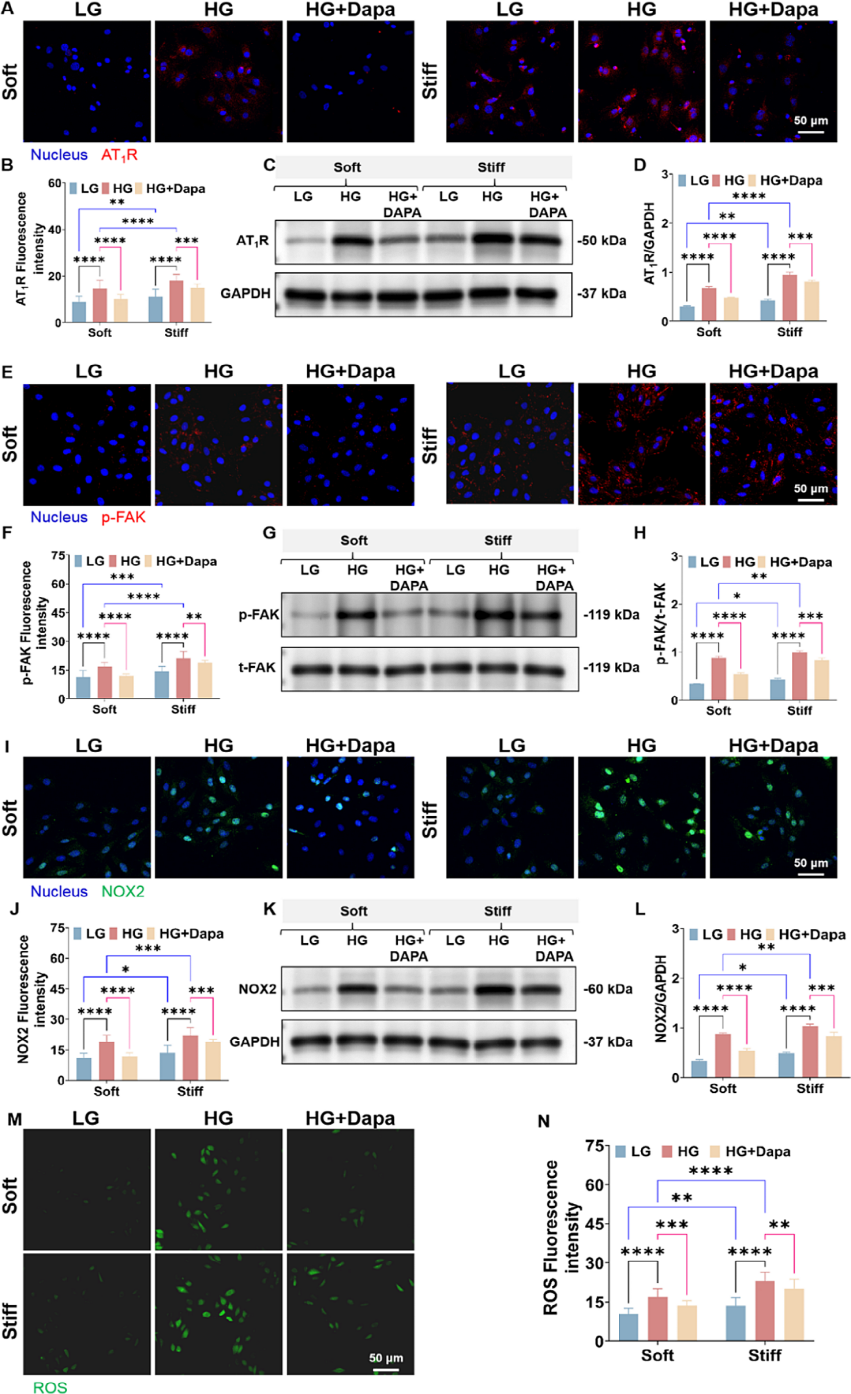
Characterization results of the AT_1_R-p-FAK-NOX2 and ROS expressions of H9c2 cells on the PA gels with stiffness of 23.9 kPa and 60.1 kPa. Fluorescence images of **A** AT_1_R, **E** p-FAK and **I** NOX2 of H9c2 cells on the PA gels with different stiffness. Statistical histogram of fluorescence intensities of **B** AT_1_R, **F** p-FAK and **J** NOX2 of H9c2 cells on the PA gels with different stiffness (*n* > 3). Western blotting analysis of **C** AT_1_R, **G** p-FAK and **K** NOX2 of H9c2 cells on the PA gels with different stiffness. The protein gray values of **D** AT_1_R, **H** p-FAK and **L** NOX2 of H9c2 cells on the PA gels with different stiffness (*n* = 3). **M** ROS fluorescence images of H9c2 cells on the PA gels with different stiffness. **N** Statistical histogram of ROS fluorescence intensities of H9c2 cells on the PA gels with different stiffness (*n*>3).*p < 0.05, **p < 0.01, ***p < 0.001, and ****p < 0.0001 determined by two-way ANOVA

The immunofluorescence staining and western blot results reveal that the expressions of AT_1_R (Fig. 4A–D) and p-FAK (Fig. 4E–H) of the high-glucose-elicited cells on the 23.9–60.1 kPa PA gels dramatically increased compared with the low-glucose groups. And compared with the cells on the 23.9 kPa PA gels, the cells on the 60.1 kPa PA gels had higher expressions of AT_1_R and p-FAK, indicating that both the stiff ECM and the high-glucose condition contribute to the increased expressions of AT_1_R and p-FAK of H9c2 cells. After the Dapa intervention under the high-glucose environment, the expressions of the mechanical signal proteins of AT_1_R and p-FAK reduced. Moreover, the significant decreases in the expressions of AT_1_R and p-FAK of the cells on the 23.9 kPa PA gels than those on the 60.1 kPa PA gels were observed. Compared to the low-glucose group, the remarkable increases in the NOX2 expressions (Fig. 4I–L) and ROS levels (Fig. 4M, N) of the cells under high glucose circumstance were obtained. Of note, the cells on the 60.1 kPa PA gels presented significantly elevated NOX2 expression and ROS level than those on the 23.9 kPa PA gels. After the Dapa treatment, there were the considerably greater decreases in the NOX2 expressions and the ROS levels of the cells on the soft PA gels than those on the stiff PA gels.

### AT_1_R-FAK-NOX2 pathway regulated the ROS level of H9c2 cells under different mechanical microenvironment

**Fig. 5:**
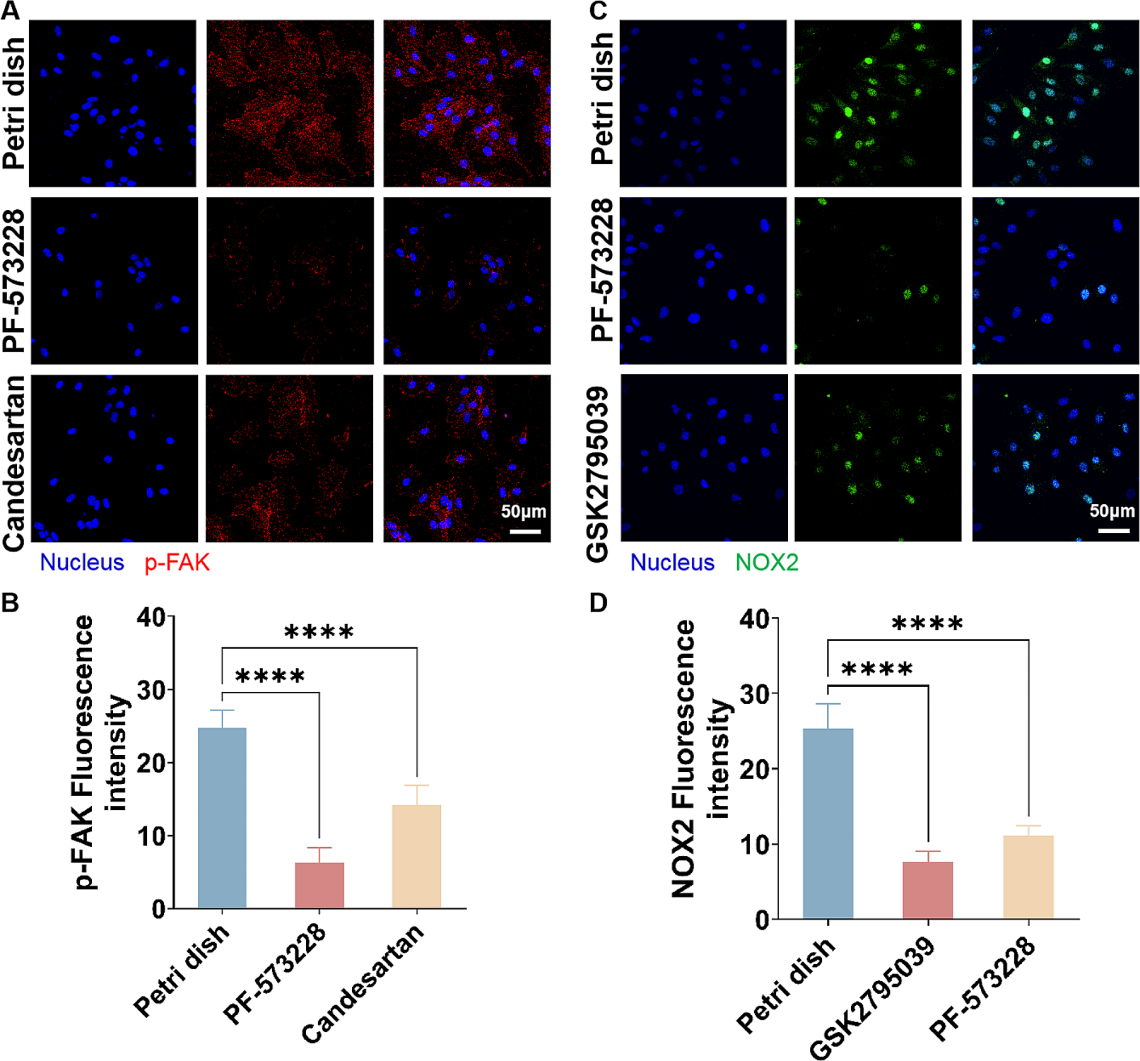
Verification the relationship of the AT_1_R-FAK-NOX2 pathway in H9c2 cells on the PA gels with stiffness of 23.9 and 60.1 kPa, respectively. **A** Fluorescence images and **B** statistical histogram of p-FAK in H9c2 cells after adding PF-753,228 and candesartan. **C** Fluorescence images and **D** statistical histogram of NOX2 in H9c2 cells after adding PF-753,228 and GSK2795039 (*n *> 3).*p < 0.05 and ****p < 0.0001 determined by two-way ANOVA

The above results indicate that both stiff ECM and high glucose conditions enhanced the AT_1_R, p-FAK and NOX2 expressions in the H9c2 cells on the PA gels. To understand the interaction of these proteins, we used PF-753,228 (a FAK inhibitor) to repress the p-FAK expression and then characterized the expression of AT_1_R, which showed that the expression of AT_1_R was not affected by the inhibition of FAK (Fig. S5A, B). In contrast, the expression of p-FAK was subsequently down-regulated with the suppressed AT_1_R expression by a AT_1_R antagonists (Candesartan), suggesting that AT_1_R was the upstream of FAK and modulated the FAK expression (Fig. 5A, B). To validate the correlation between FAK and NOX2, we used a NOX2 inhibitor (GSK2795039) to restrain the NOX2 expression. The immunofluorescence results of NOX2 and p-FAK in Fig. S5C, D reveal that inhibition of NOX2 had no effect on the p-FAK expression. However, the NOX2 expression was vigorously downregulated with the FAK inhibitor (PF-753,228), indicating that FAK can positively regulate NOX2 (Fig. 5C, D). These results confirm that AT_1_R could promote NOX2 by activating FAK phosphorylation. Finally, to verify the effect of ROS level on NOX2 expression, we treated the cells with an antioxidant (N-Acetyl-L-cysteine, NAC) and characterized the NOX2 protein level via immunofluorescence, unveiling that the addition of NAC did not affect the NOX2 expression (Fig. S5E, F). Moreover, the ROS levels in cells presented noticeable reduction after adding both NAC and the NOX2 inhibitor (Fig. S6), indicating that ROS was under the regulation of NOX2. Based on these results, we can clarify the upstream and downstream relationships of the AT_1_R-FAK-NOX2 pathway, which had the regulatory effect on the ROS levels of cells.

### Effect of Dapa on the expressions of integrin β1 and piezo 1 in H9c2 cells under different mechanical microenvironment

**Fig. 6:**
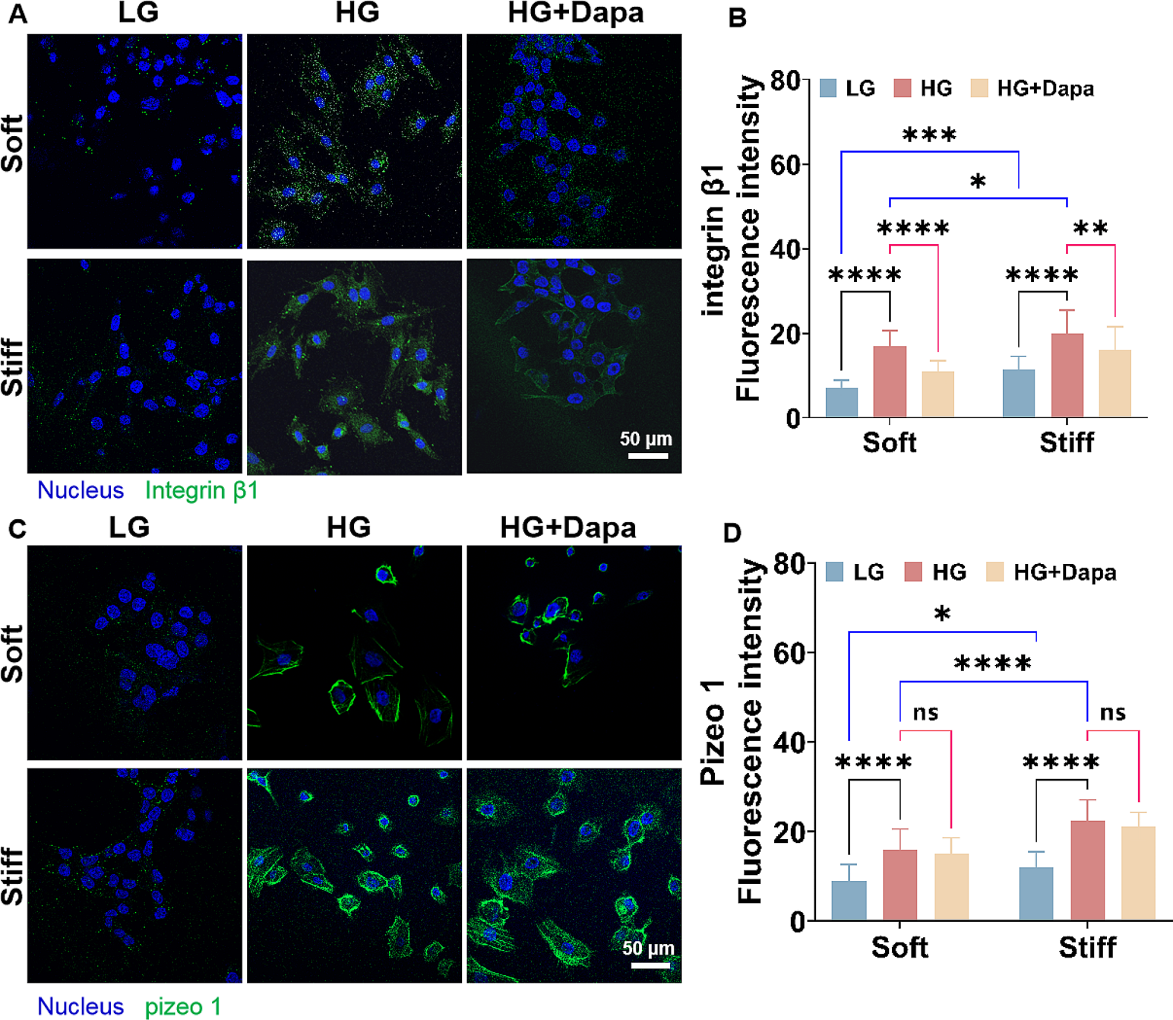
Characterization results of integrin β1 and piezo 1 expressions in H9c2 cells on the PA gels with stiffness of 23.9 and 60.1 kPa. **A** Fluorescence images and **B** statistical histogram of integrin β1 in the H9c2 cells on the PA gels with different stiffness (*n*>3). **C** fluorescence images and **D** statistical histogram of piezo 1 in H9c2 cells on the PA gels with different stiffness (*n*>3). ns, no significant difference, *p < 0.05, **p < 0.01, ***p < 0.001, and ****p < 0.0001 determined by two-way ANOVA

Considering that integrin β1 and piezo 1 are two key cardiac mechanotransducers for maintaining normal function of heart, we also characterized the expressions of these two mechanical signaling proteins after Dapa intervention. The immunofluorescence analysis of integrin β1 demonstrated that the high glucose condition enhanced the expressions of integrin β1 in the H9c2 cells on both 23.9 and 60.1 kP PA gels. Moreover, compared to those on the soft PA gels, H9c2 cells exhibited higher integrin 1 expressions on the stiff PA gels (Fig. 6A, B). While, the integrin β1 expression presented a distinct reduction after the Dapa treatment. And a greater reduction in the integrin β1 expression in the H9c2 cells on the soft PA gels than those on the stiff PA gels was observed. In addition, the high glucose environment induced the increased piezo 1 expression in the cells, which was more obvious in the cells on the stiff PA gels (Fig. 6C, D). However, no significant alteration in the piezo 1 expression in the H9c2 cells after Dapa treatment was obtained, suggesting that Dapa did not exert a direct impact on the expression of piezo 1.

## Discussion

Long-term hyperglycemia would result in the increased stiffness of cardiac ECM, promoting fibrosis and causing pathological changes in DCM [24]. Dapa has been applied globally for treating type 2 diabetes and the positive efficacy of Dapa in cardiovascular disease recommends the SGLT2 inhibitors in HFrEF treatment in the international heart failure guidelines [25, 26]. Previous studies have unveiled that Dapa has beneficial effects on DCM through multiple pathways [27]. For example, it can alleviate the damage of hyperglycemia to myocardial cells and prevent further deterioration of myocardial cell function via attenuating blood glucose levels [28]. In addition, Dapa has been proven to be capable to reduce the inflammation and fibrosis (the two key features of DCM) through inhibiting the generation of inflammatory factors and accumulation of ECM [28–31]. Even the impacts of ECM stiffness on DCM progression and Dapa’s regulation of mechanical signaling proteins are still not fully clear, our study shows that Dapa can therapeutically affect DCM via inhibiting the expressions of mechanical signaling proteins under different ECM stiffness. And the collagen deposition, myocardial injury, oxidative stress in the DCM rats and the DCM progression can be reduced by Dapa via inhibiting the AT_1_R-FAK-NOX2 pathway in myocardial tissues. Both the in vitro and in vivo experimental results demonstrate that Dapa exhibited better therapeutic efficacy in DCM under softer ECM compared to the stiffer ECM, indicating the significant role of ECM stiffness in Dapa’s efficacy.

Currently, the rapid development of cardiac tissue engineering offers distinct chances for cardiovascular disease modeling, drug testing and regenerative medicine applications [32, 33]. In the in vitro cardiac models, ECM provides a unique physical microenvironment (physiological or pathological) for resident cardiac cells, and the cardiac cell models with different ECM stiffness can be constructed via PA gels with adjustable stiffness [34, 35]. Previous studies have shown that excessive matrix deposition and tissue stiffening are the key pathological features in cardiovascular diseases, especially myocardial fibrosis [36–38], which can be due to that abnormal matrix stiffness influences the functions of myocardial cells and cardiac fibroblasts (e.g., proliferation, migration and hypertrophy) and leads to the emergence and progression of cardiovascular diseases [39, 40]. For instance, Walker et al. found that ECM stiffness could modulate the activation of valvular interstitial cells through epigenetic remodeling [41]. In our work, we found that the DCM rats in the 16-weeks group with stiffer cardiac tissues and higher mechanical signal transduction protein expressions had worse cardiac function and higher oxidative stress than those in the 8-weeks group. And Dapa improved the cardiac function and reduced the oxidative stress, with more significant benefits observed in the 8-weeks group, highlighting the difference in the Dapa’s efficacy based on the heart tissue stiffness. Similar phenomena were also observed in the in vitro cell models, that is, on the stiff ECM (e.g., 60.1 kPa PA gels), Dapa showed better efficacy to reduce the cellular damage and the oxidative stress induced by high glucose compared to those on the soft ECM (e.g., 23.9 kPa PA gels). The decreased myocardial cell viability under stiff ECM condition can be attributed to multiple factors, including the changes in the cellular morphology, the interplay between cell-ECM and cellular structure, which can indirectly or directly affect the function, signaling, and coordination of the myocardial cells and then culminating in attenuated cellular viability [42]. So the dynamic adaptation process of myocardial cells to the changes in the ECM stiffness of the surrounding microenvironment can lead to the distinctions in the biological functions of myocardial cells, which results in the different drug responsiveness of myocardial cells. And it is also noted that the overall stiffness of the cardiac tissue does not directly equate to the stiffness of its ECM alone. But considering that the myocardial cells are not isolated but embedded within the intricate ECM, we assessed the stiffness of the myocardial tissue specimen to reflect the responses of the myocardial cells to Dapa efficacy under different ECM stiffness.

Cells interact with their surrounding ECM, sense and respond to the physical properties of ECM (e.g., stiffness and tension) via multiple mechanical signaling pathways such as integrin, AT_1_R, and piezo 1 [43, 44]. It has been proven that the abnormal expressions of integrin, AT_1_R, and piezo 1, the important mechanical signal proteins on myocardial cell membrane, are closely related to the cardiovascular diseases. For example, integrin β1, as the most common β subunit in the integrin family, forms various integrin heterodimers with different α subunits to modulate cell adhesion, migration, and signal transduction processes [45]. It has been reported that integrin β1 plays a key role in fibrogenic conversion of cardiac fibroblast via directly stimulating FAK/Src cascades or accentuation of growth factor signaling [46]. AT_1_R is a subtype of angiotensin II receptors, associated with perception of matrix stiffness and cellular function [43]. According to the previous report [47], the binding of angiotensin to AT_1_R can promote the association of scaffolding proteins (e.g., paxillin and talin) and lead to the focal adhesion and extracellular matrix formation. Moreover, the mechanically activated piezo1 channels can convert mechanical signals of myocardial cells into Ca^2+^ and ROS signals, which critically determine the mechanical activity of heart and the specific knockout of piezo1 in mouse heart can lead to the defects in Ca^2+^ and ROS signaling and the development of cardiomyopathy [48, 49]. In this work, we found that the stiff ECM could enhance the expressions of integrin β1, AT_1_R, and piezo 1, and the Dapa treatment could reduce the expressions of integrin β1 and AT_1_R but without an obvious impact on the expression of piezo1. Previous studies showed that AT_1_R plays a vital role in most cardiovascular diseases, and the specific blocking of AT_1_R can prevent or reduce the diabetes-related cardiovascular complications [50, 51]. And the activation of AT_1_R in cardiovascular system is related to the pathological processes such as cardiac hypertrophy, fibrosis, inflammatory response, and oxidative stress [52]. Therefore, we selected AT_1_R as the research target and clarified the role of AT_1_R in the cell perception of ECM stiffness under DCM condition, providing the therapeutic references for the DCM treatment.

The experiment results of immunohistochemistry staining demonstrated that compared to the normal rats, the expressions of AT_1_R in the myocardial tissues of the DCM rats were greatly higher, thus the excessive activation of AT_1_R under high glucose condition can contribute to the cell hypertrophy and myocardial fibrosis [53]. In addition, we found that the expressions of p-FAK and NOX2 in the myocardial tissues of the DCM rats also significantly increased compared to those of the normal rats. According to the previous literature [54], FAK is a key mechanical signal transduction protein in the cytoplasm of myocardial cell and its increased phosphorylation expressions in the myocardial cells are regulated by membrane proteins related to the mechanical transduction, which lead to the increased cardiac load, high glucose, and inflammation. NOX2 is a subunit of NADPH oxidase and participates in the oxidative stress in the myocardial tissues. Thus, we validated the relationship between AT_1_R, FAK, and NOX2 through cell experiments to mimic the DCM condition. The experimental results showed that the high glucose and the increased ECM stiffness could cause the increased expressions of membrane protein AT_1_R to initiate the p-FAK signaling and thereby upregulate the NOX2 expression, which can increase the oxidative stress in myocardial cells through production of excessive ROS and trigger the inflammatory reaction and cell damage [55]. Dapa can improve the heart function and reduce the myocardial cell damage in the DCM rats via inhibiting the AT_1_R-FAK-NOX2 signaling pathway. Thus, controlling the AT_1_R activation and inhibiting the p-FAK/NOX2 signaling pathway can be beneficial to the prevention and treatment of DCM, providing the inspiration for further study of the pharmacological mechanisms of Dapa. And the most recent 2021 guidelines for the heart failure treatment published by the European Society of Cardiology also recommends Dapa as a first-line treatment for heart failure patients with decreased ejection fraction [56]. And the growing evidence has established that Dapa has a clinically meaningful reduction in cardiovascular disease mortality, clinical events, and quality of life [57, 58]. Our experimental results showed that after Dapa intervention, the degrees of cardiac function deterioration in the 8-weeks group of rats were obviously lower compared to those in the 16-weeks group of rats, indicating that Dapa intervention at the early stage of DCM can better delay the further deterioration of DCM than the Dapa intervention at the late stage of DCM. The previous clinical trials also showed that the great clinical benefits of SGLT2 inhibitors occurred early, such as from days to weeks after treatment [59]. Our study elucidates the therapeutic effect of Dapa on DCM through the AT_1_R-FAK-NOX2 signaling pathway. We can thus infer that the early Dapa intervention for T2D patients or DCM patients can delay the occurrence of diabetes complications and the DCM cardiovascular events. However, a key obstacle to the development of effective drugs to treat DCM is the complexity and redundancy of fibrosis signal transduction. An underrecognized reason for this complexity is the synergistic interaction between biochemical and mechanical regulation. Hence, it is still needed to further explore the mechanism of these signaling pathways in the pathological process of DCM.

### Limitation

Our work indicates that the ECM stiffness modulates the AT_1_R-FAK signaling pathway and thereby impacts the Dapa’s efficacy in DCM. But it is also important to concern other factors contributed to the Dapa’s efficacy. For example, the genetic diversity among rat model, the inflammatory status and the difference in the metabolic level of rats all might individually or collectively influence the Dapa’s efficacy, which can be performed in our future study for developing the targeted therapeutic approaches for diabetic cardiomyopathy.

## Conclusion

In this work, we proposed that that Dapa can diminish the occurrence and progression of DCM via ameliorating heart function and reducing oxidative stress levels through inhibiting the AT_1_R-FAK-NOX2 signaling pathway in the myocardial tissues. The occurrence and progression of DCM were accompanied by the increased expressions of mechanical signal transduction proteins, and Dapa could inhibit the increases of mechanical protein expressions and thus contribute to the DCM treatment. More importantly, the early Dapa intervention for T2D patients or DCM patients can delay the occurrence of diabetes complications and DCM cardiovascular events. This is the first report to investigate the correlation between ECM stiffness and Dapa efficacy on DCM that is a new discovery in the pharmacological study of Dapa. The finding of this work can attribute to a better understanding of the ECM stiffness impact on the progress and treatment of DCM and also emphasizing the physiological effect of ECM stiffness on the drug efficacy in clinically treating DCM with proposing that the AT_1_R-FAK-NOX2 signaling pathway can be a new target for intervention of ECM stiffness in DCM.

### Supplementary material

Supplementary Material 1. Figure S1. Photos and weights of cardiac anatomy of the DCM rats without and with Dapa treatment; Figure S2. Young’s modulus of the as-prepared PA gels; Figure S3. Young’s modulus of (A) the soft PA gels and (B) stiff PA gels before and after seeding of H9c2 cells; Figure S4. Statistical histogram of the H9c2 cells viability on the PA gels with stiffness of 23.9 and 60.1 kPa; Figure S5. Analysis and verification of the AT_1_R-FAK-NOX2 pathway in H9c2 cells on the PA gels with stiffness of 23.9 and 60.1 kPa; Figure S6. Fluorescence images and statistical histogram of fluorescence intensities of ROS of H9c2 cells on the PA gels with stiffness of 23.9 and 60.1 kPa after adding GSK2795039 (a NOX2 inhibitor) and NAC.

## Data Availability

No datasets were generated or analysed during the current study.

## Abbreviations

AGEs: Advanced glycation end products
AT_1_R: Angiotensin II Type 1 Receptor
BNP: Brain natriuretic peptide
C-TnT: Cardiac troponin T
Dapa: Dapagliflozin
DCM: Diabetic cardiomyopathy
ECM: Extracellular matrix
EF: Ejection fraction
GK rats: Goto-Kakizaki Rats
HFrEF: HF with reduced ejection fraction
FS: Fractional shortening
IVSTd: Interventricular septum thickness at end-diastole
LVIDd: Left ventricular internal diameter at end-diastole
PA: Polyacrylamide
ROS: Reactive oxygen species
SGLT2: Sodium-glucose co-transporter-2
T2D: Type 2 diabetes
