# Supplementary material for: Effect of extracellular matrix stiffness on efficacy of Dapagliflozin for diabetic cardiomyopathy
Source: Cardiovasc Diabetol. 2024 Jul 24;23:273. doi: 10.1186/s12933-024-02369-x (PMC11270890; doi:10.1186/s12933-024-02369-x)
Supplement: Supplementary file 1 — Supplementary Material 1. Figure S1. Photos and weights of cardiac anatomy of the DCM rats without and with Dapa treatment; Figure S2. Young’s modulus of the as-prepared PA gels; Figure S3. Young’s modulus of (A) the soft PA gels and (B) stiff PA gels before and after seeding of H9c2 cells; Figure S4. Statistical histogram of the H9c2 cells viability on the PA gels with stiffness of 23.9 and 60.1 kPa; Figure S5. Analysis and verification of the AT1R-FAK-NOX2 pathway in H9c2 cells on the PA gels with stiffness of 23.9 and 60.1 kPa; Figure S6. Fluorescence images and statistical histogram of fluorescence intensities of ROS of H9c2 cells on the PA gels with stiffness of 23.9 and 60.1 kPa after adding GSK2795039 (a NOX2 inhibitor) and NAC. [file 12933_2024_2369_MOESM1_ESM.doc]

**Additional File**

**Effect of extracellular matrix stiffness on efficacy of Dapagliflozin for diabetic cardiomyopathy**

Tong Zhu^1,2,3^, Zhaoyang Ye^1,2^, Jingjing Song^1,2^, Junjie Zhang^1,2^, Yuxiang Zhao^1,2^, Feng Xu^1,2^, Jun Wang^4^, Xin Huang^5^, Bin Gao^6^*, Fei Li^1,2^*

*^1^ The Key Laboratory of Biomedical Information Engineering of Ministry of Education, School of Life Science and Technology, Xi’an Jiaotong University, Xi’an 710049, P.R. China*

*^2^ Bioinspired Engineering and Biomechanics Center (BEBC), Xi’an Jiaotong University, Xi’an 710049, P.R. China*

*^3^ Department of Cardiovasology, Xidian Group Hospital, Xi’an 710077, P.R. China*

*^4^ Department of Health Evaluation and Promotion, The First Affiliated Hospital of Xi'an Jiaotong University, Xi’an 710061, P.R. China*

*^5^ Department of Cardiology, The First Affiliated Hospital of Xi'an Jiaotong University, Xi'an, 710061, P.R. China*

*^6^ Department of Endocrinology, Tangdu Hospital, Air Force Military Medical University, Xi’an 710032, P.R. China*

**Corresponding authors: bingao@fmmu.edu.cn; feili@mail.xjtu.edu.cn*

**Additional Figures**


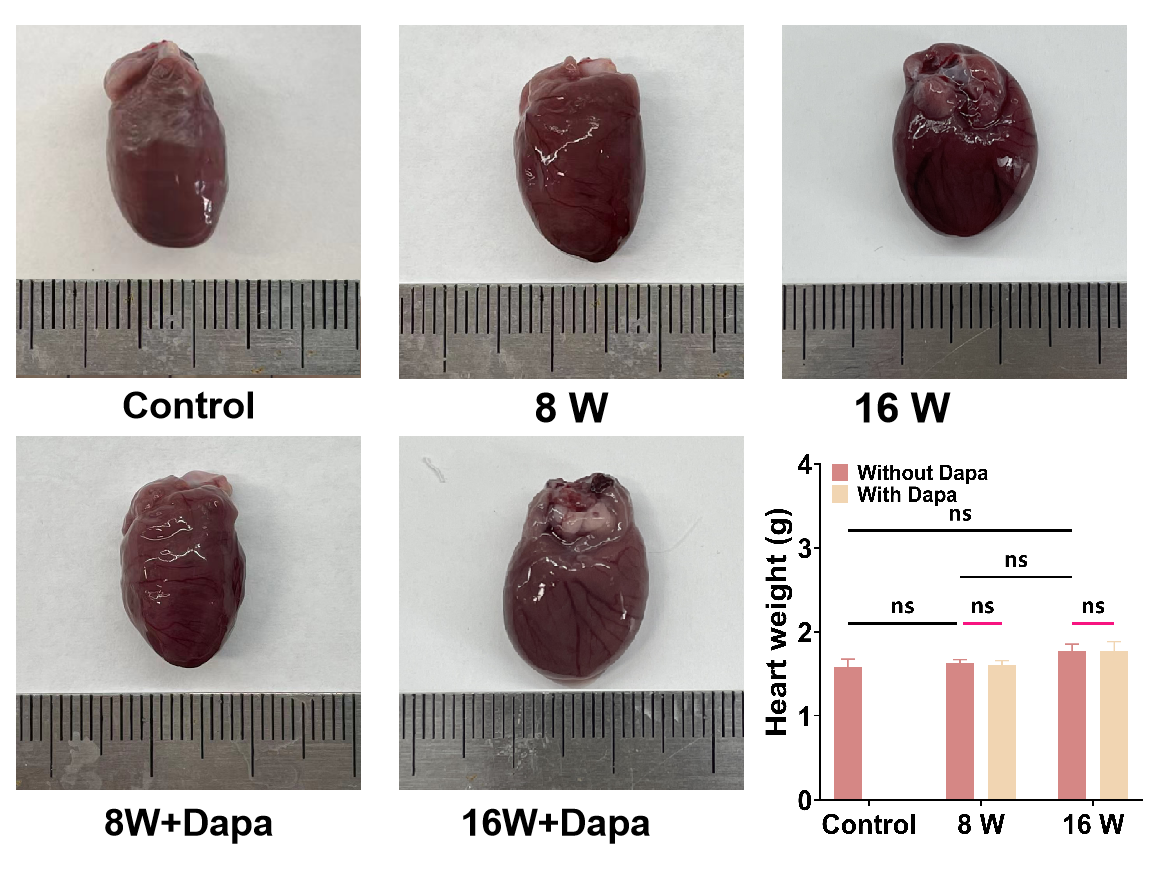


**Figure S1. Photos and weights of the cardiac anatomy of the DCM rats without and with Dapa treatment (*n* = 4)**. Data are shown as means±SEM. ns, no significant difference.


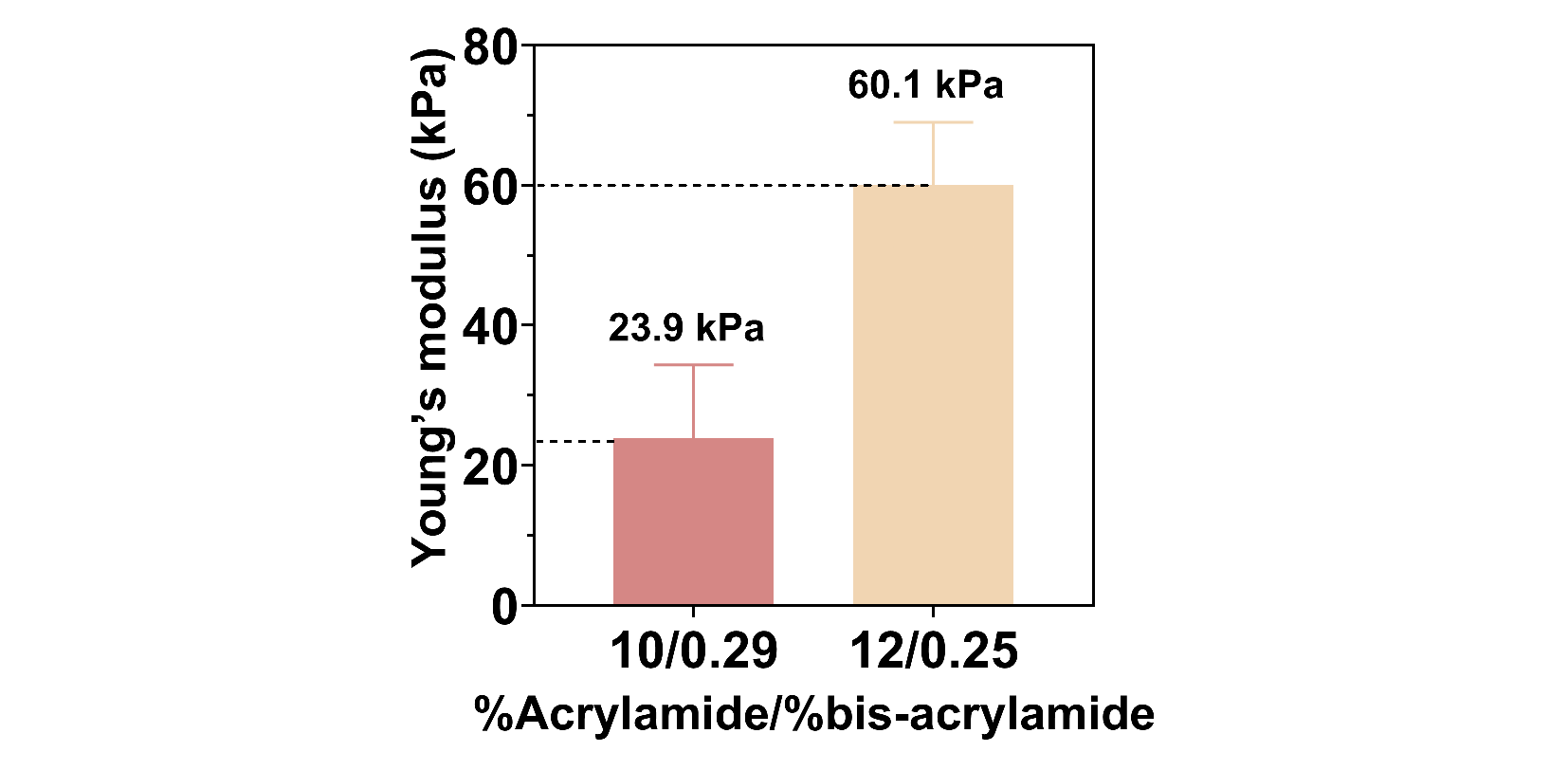


**Figure S2.** **Young’s modulus of the as-prepared PA gels (*n*＞3)**.


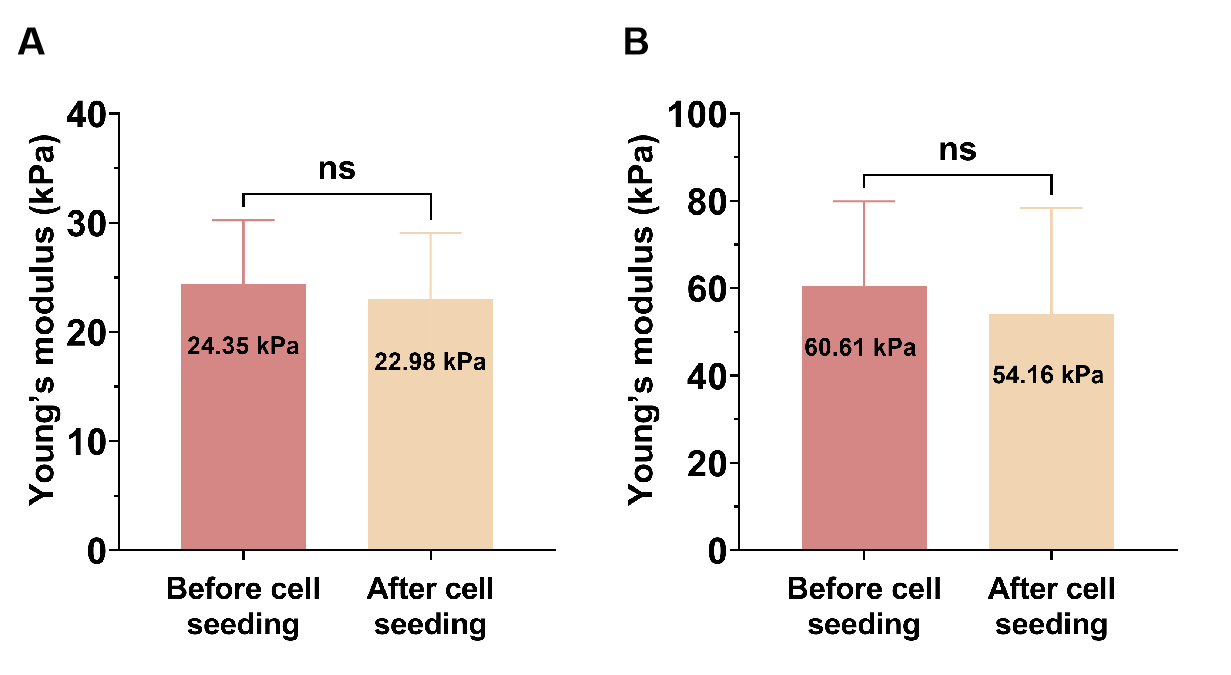


**Figure S3.** **Young’s modulus of (A) the soft PA gels and (B) stiff PA gels before and after seeding of H9c2 cells**.


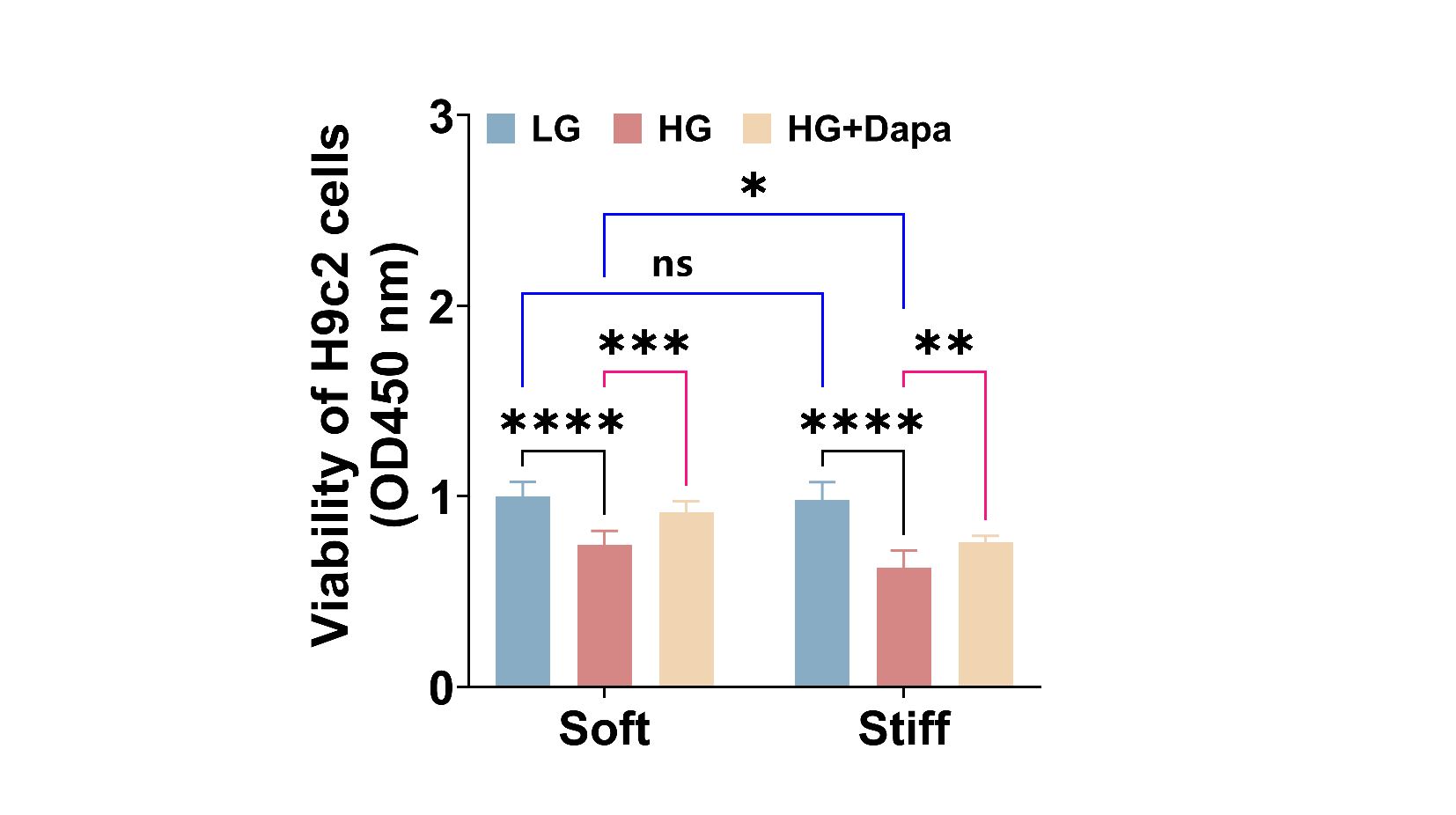


**Figure S4.** **Statistical histogram of the H9c2 cells viability on the PA gels with stiffness of 23.9 and 60.1 kPa**. ns, no significant difference, **p*<0.05, ***p*<0.01, ****p*<0.001, and *****p*<0.0001 determined by two-way ANOVA.


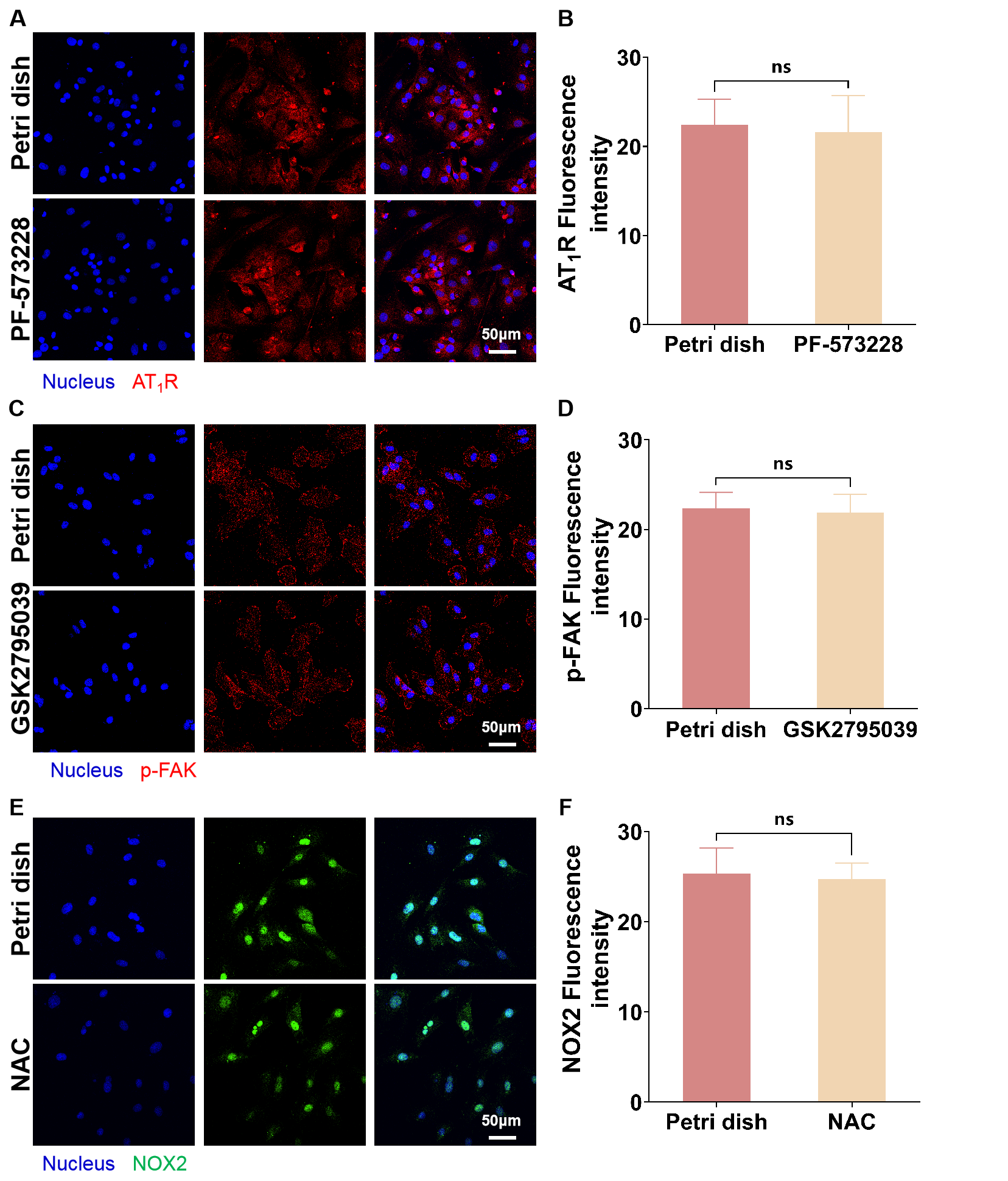


**Figure S5. Analysis and verification of the AT_1_R-FAK-NOX2 pathway in H9c2 cells on the PA gels with stiffness of 23.9 and 60.1 kPa.** (A) Fluorescence images and (B) statistical histogram of fluorescence intensities of AT_1_R of H9c2 cells on the PA gels after adding a FAK inhibitor (PF-573228). (C) Fluorescence images and (D) statistical histogram of fluorescence intensities of p-FAK of H9c2 cells on the PA gels after adding a NOX2 inhibitor (GSK2795039). (E) Fluorescence images and (F) statistical histogram of fluorescence intensities of ROS levels of H9c2 cells on the PA gels after adding NAC (*n*＞3). ns, no significant difference.


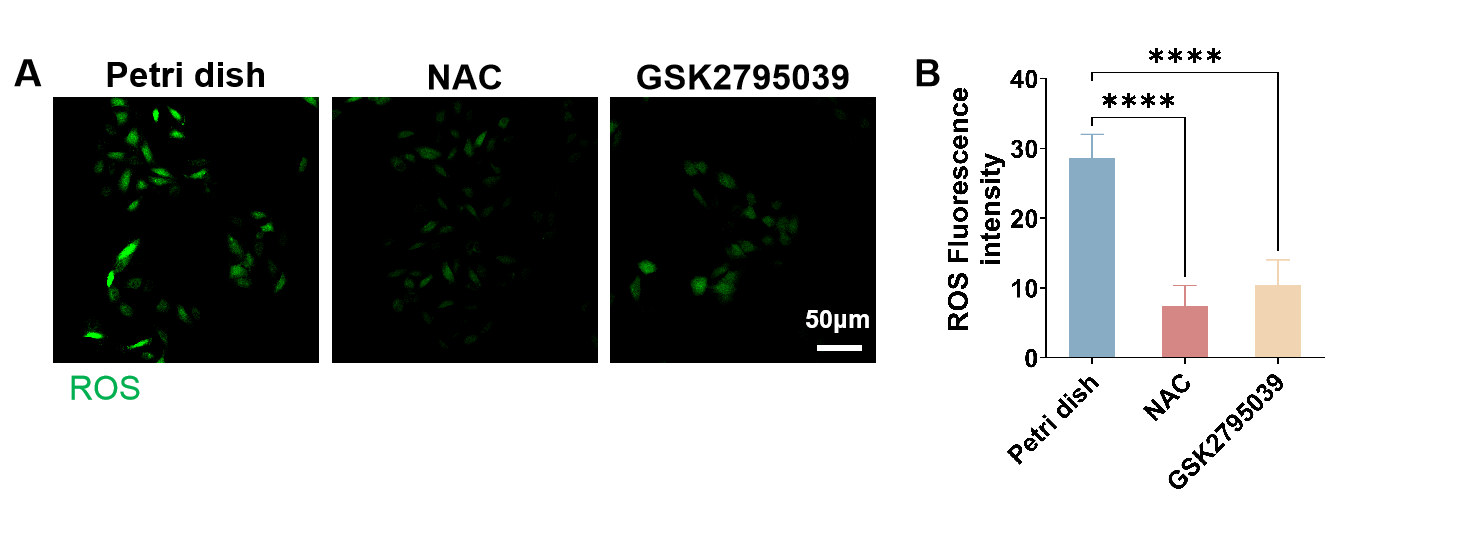


**Figure S6.** (A) Fluorescence images and (B) statistical histogram of fluorescence intensities of ROS of H9c2 cells on the PA gels with stiffness of 23.9 and 60.1 kPa after adding GSK2795039 (a NOX2 inhibitor) and NAC (*n*＞3). *****p*<0.0001 determined by two-way ANOVA.
